# Evaluation of Locally Available Detergents for Detection of Subclinical Mastitis in Ugandan Dairy Cattle

**DOI:** 10.1002/vms3.70635

**Published:** 2025-10-21

**Authors:** Denis Rwabiita Mugizi, Benjamin Kulaaza, Patrick Arthur Kalibbala, Gerald Nizeyimana, Paul Ssuna

**Affiliations:** ^1^ College of Veterinary Medicine Animal Resources and Biosecurity Makerere University Kampala Uganda

**Keywords:** California Mastitis Test, detection, mastitis, washing detergents

## Abstract

Mastitis remains a major production constraint of dairy cattle farmers in Uganda leading to reduced production. Subclinical mastitis, though often ignored, is more prevalent and causes more economic losses compared to clinical mastitis. Studies have shown a consistently high prevalence of subclinical mastitis in different areas of Uganda. Screening is crucial in the control of subclinical mastitis but the most used screening test, the California Mastitis Test, is rather expensive and widely inaccessible to most poor farmers in Uganda. Research has shown that laundry detergents can accurately screen for subclinical mastitis which would be convenient for farmers in Uganda due to their cheapness and availability country wide. The aim of this study was to establish the prevalence of subclinical mastitis in dairy cattle in Kampala, the cow level risk factors and to determine the effectiveness of locally available domestic detergents in screening for subclinical mastitis. The study was carried out on 140 randomly selected lactating cattle in urban and peri‐urban Kampala, Uganda. The prevalence of subclinical mastitis was determined using the California Mastitis Test (CMT). Optimized concentrations of the different detergent solutions were then tested using the same procedure as the CMT and their scores based on the gel formation of reagent with milk were recorded. A cow with at least one quarter with score ≥ 1 was considered positive for subclinical mastitis. The prevalence of subclinical mastitis was 81.6%. Parity and breed were significantly associated with subclinical mastitis. Primiparous cows were less susceptible (Odds ratio = 0.189; 95% CI: 0.069–0.521) as compared to multiparous. Friesians were more susceptible to subclinical mastitis (Odds ratio = 8.047; 95% CI: 1.268–51.078) compared to the local breeds. The Fleiss kappa, *k* = 0.556 (95% CI: 0.504–0.608, *p* < 0.001) in the ability to detect subclinical mastitis was seen in all detergents tested indicating a moderate to substantial reliability of the detergents when compared with the commercial CMT reagent. The area under the receiver operating characteristics curve (AUC) of the detergents as compared to the CMT, ranged from 0.814 to 0.905 implying a high to excellent accuracy. Six (6%) magic detergent showed the best performance (AUC: 0.905) of all the locally available domestic detergents used in Uganda. This showed that local domestic detergents can be used as an alternative to the expensive and inaccessible CMT to detect subclinical mastitis in Uganda. This study therefore recommends veterinary legislation on the usage of domestic detergents in screening for subclinical mastitis and a willingness study by farmers in Uganda to uptake this diagnostic procedure.

## Graphical abstract

This study established that mastitis is highly prevalent in cattle in Kampala, Uganda and locally available washing detergents like Magic, Ariel, Sunlight and OMO have a comparable diagnostic accuracy of subclinical mastitis compared to commercial California Mastitis Test (CMT) reagent. These detergents could offer alternatives to CMT in mastitis screening.

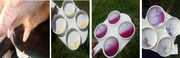

## 1 Introduction

Mastitis is a very costly disease in dairy production accounting for up to 70% of avoidable milk losses worldwide (Kher et al. 2018). Although clinical mastitis is severe and debilitating to the dairy cattle, as the signs of disease are easily seen, treatment usually follows promptly. However, the hidden sub clinical form, which is more prevalent, causes greater economic losses oblivious to farmers (Hasan et al. 2018). Although the loss due to subclinical mastitis is difficult to quantify, studies have shown that it costs the average dairy farmer more than the clinical mastitis does (Huijps et al. 2008). Although there is no nationwide study on prevalence of sub clinical mastitis in Uganda, research in different areas of the country can give us a vague idea on the rather alarmingly high prevalence of mastitis in the country. The prevalence in urban and peri‐urban Kampala was found to be at 86.2% (Abrahmsén et al. 2014) whereas another study put the figure at 90% (Björk 2013); in Kiboga District was at 87.9% (Tingiira and Vudriko 2014). The prevalence in Jinja district was at 61.3% (Byarugaba 2008) while in Kiruhura district, 76.1% of cattle tested had mastitis although the type of mastitis was not differentiated (Ssajjakambwe et al. 2017).

The best way of reducing mastitis in dairy cows is by enforcing proper control measures to improve hygiene of cows, milk machines and milking personnel (Degraves and Fetrow 1993; Mcdougall 2011). It is also paramount to detect cows early for mastitis and treatment before spreading to other cows in the herd. The gold standard of diagnosing mastitis is by bacterial culture. This however is expensive and impractical to be done in the field (Viguier et al. 2009). The California Mastitis Test (CMT) is the most used screening test worldwide because it is relatively in expensive and fairly accurate compared to bacteria culture (Sharma et al. 2010). However, in Uganda, one of the poorest countries in the world, with 34.6% of households living below the international extreme poverty line of $1.90 a day (World Bank 2016), the seemingly cheap cost of the CMT is simply not affordable by many smallholder farmers in the country. Furthermore, the CMT reagent is not readily available countrywide for the farmers who could afford it. Several detergent based mastitis screening reagents have been developed in some countries among which is the Surf Field mastitis Test (SFMT) reagent (Muhammad et al. 2010), the Ethiopia mastitis test reagent (Zeweld and Tarekegn 2025), sodium lauryl sulphate mastitis test reagent (Tanni et al. 2021) and a local mastitis test reagent (Sihhaila et al. 2021). All these reagents have shown comparable diagnostic potential of subclinical mastitis like the CMT. The SFMT, basically a 3% detergent solution, has shown similar sensitivity and specificity as compared to the CMT (Muhammad et al. 2010). A similar study in Uganda using locally available detergents which are very cheap and available countrywide could yet be a key step in controlling subclinical mastitis in herds in Uganda. The Surf Field, Ethiopian mastitis test and sodium lauryl sulphate reagents though available in some African countries are not readily available in Uganda, thus evaluating the diagnostic potential of locally available washing detergents could help overcome this challenge. Detergents are known to pose chemical and packaging challenges to the environment, but the amounts used in subclinical mastitis screening is small and with proper packaging in biodegradable bags and appropriate disposal of the residues, detergents can provide an alternative to CMT reagent.

## 2 Materials and Methods

### 2.1 Study Area

The study was carried out in urban and peri‐urban Kampala District. Kampala city is the capital and largest city in Uganda, with an area of 189 km^2^. The city is divided into five Divisions: Kampala Central Division, Kawempe Division, Makindye Division, Nakawa Division and Rubaga Division. According to the 2008 National Livestock Census, there are 32,000 heads of cattle which are predominantly dairy cows. The report further states that about 7000 households out of the estimated 390,000 in urban and peri‐urban Kampala have at least one cow (MAAIF 2009).

### 2.2 Study Design

This was an experimental and cross‐sectional study design that was done between May and June 2018 to determine the prevalence of subclinical mastitis in urban and peri‐urban Kampala using the CMT. The cow level risk factors were recorded for each individual cow. After carrying out the CMT test, the cows were subjected to tests with locally available detergents in the same procedure as that of the CMT.

### 2.3 Sample Size Determination and Sampling Strategy

The sample size (*N*) for the study was determined using a formula by Malhotra and Indrayan (2010) that compares sensitivity of tests at a given prevalence of a condition.

$$N=\frac{Z^{2}Se\left(1-Se\right)}{d^{2}\mathrm{Prev}}$$

Where Prev is the expected prevalence subclinical mastitis (90%), *S*_e_ is the anticipated sensitivity of the detergent test which was 91% in a previous study (Shahid et al. 2011); *Z* is the standard normal deviate corresponding to the specified size of the critical region taken as 1.96 and, *d* is absolute precision taken at 5%. This formula when computed gave a sample size of 140 cows. A list of herds was generated with the help of an Artificial Inseminator working in urban and peri‐urban Kampala. The farmers referred the researcher to other farmers who had lactating cows, and this was an easier way to locate and include herds with lactating animals since there was no milk collection centre in the area that would be used to identify farms with lactating animals. However, snow balling technique introduces bias.

### 2.4 Clinical Inspection of Udder for Clinical Mastitis

The udder was first examined visually and then through palpation to detect clinical mastitis. Signs of swelling, pain, heat of the udder were examined alongside swelling of the supramammary lymph nodes. Milk secretions from each mammary quarter were examined for the presence of clots, flakes, blood and watery secretions (Abera et al. 2014; Biffa et al. 2005).

### 2.5 Screening for Subclinical Mastitis

A four‐compartment paddle was introduced below the udder of a milking cow after fore stripping. Approximately 3 mL of milk was drawn from each quarter onto the paddle. An equal volume of CMT reagent was added, followed by gentle rotation of the paddle for a few seconds. The results were read, and the scores recorded. A score of CMT ≥ 1 based on the gel formation was considered positive for subclinical mastitis (Kivaria and Kapaga 2004). The borderline scores between the score of 0 and +1 were considered as negative and given a score of 0.

### 2.6 Local Domestic Detergents

Since the concentrations of anionic surfactants in the different brands of domestic detergents are not readily available due to issues of trade secrets and patents, optimization of the concentrations was based on the 3% solution of the SFMT and 3 % sodium lauryl sulphate which is a component in most washing detergents and has been tested and found to be effective in screening for subclinical mastitis (Bachaya et al. 2011;Tanni et al. 2021). Solutions varying from 2% and 7% were prepared with Rwenzori mineral water to find the optimum concentration of each detergent to be used tested against laboratory confirmed cows with subclinical mastitis. The chosen concentrations were chosen in such a way that they close to the concentrations of already tested detergent based mastitis screening reagents like 3% surf field reagent and 3% sodium lauryl sulphate, which are components in most locally available detergents (Tanni et al. 2021). OMO, ARIEL, SUNLIGHT and MAGIC detergents were used. The solutions were prepared by mixing the detergent powder ranging from 2 to 7 g in 100 mL of water and tested against laboratory confirmed positive cows for subclinical mastitis. This resulted into optimized concentrations solutions of 6% Omo, 5% Ariel, 5% Sunlight and 6% Magic solutions.

For each of the selected cows, the udder and teats were cleaned with warm water and dried with a towel and fore stream milk stripped onto a strip cup to ensure that cows in the study were not having clinical mastitis. Mid‐stream milk per teat was then milked onto separate wells of the California mastitis screening peddle and equilibrated to two (2) mL per well by tilting the peddle until the milk reached a 2 mL mark on each peddle. The milk was then mixed with equal amounts of the four washing detergent solutions and the paddle swirled horizontally for 15 s and the gel formation ranked on a score similar to that of the CMT (Muhammad et al. 2010). The procedure was repeated for the four washing detergents and the CMT reagent for each of the cows being screened.

### 2.7 Data Analysis

**FIGURE 1:**
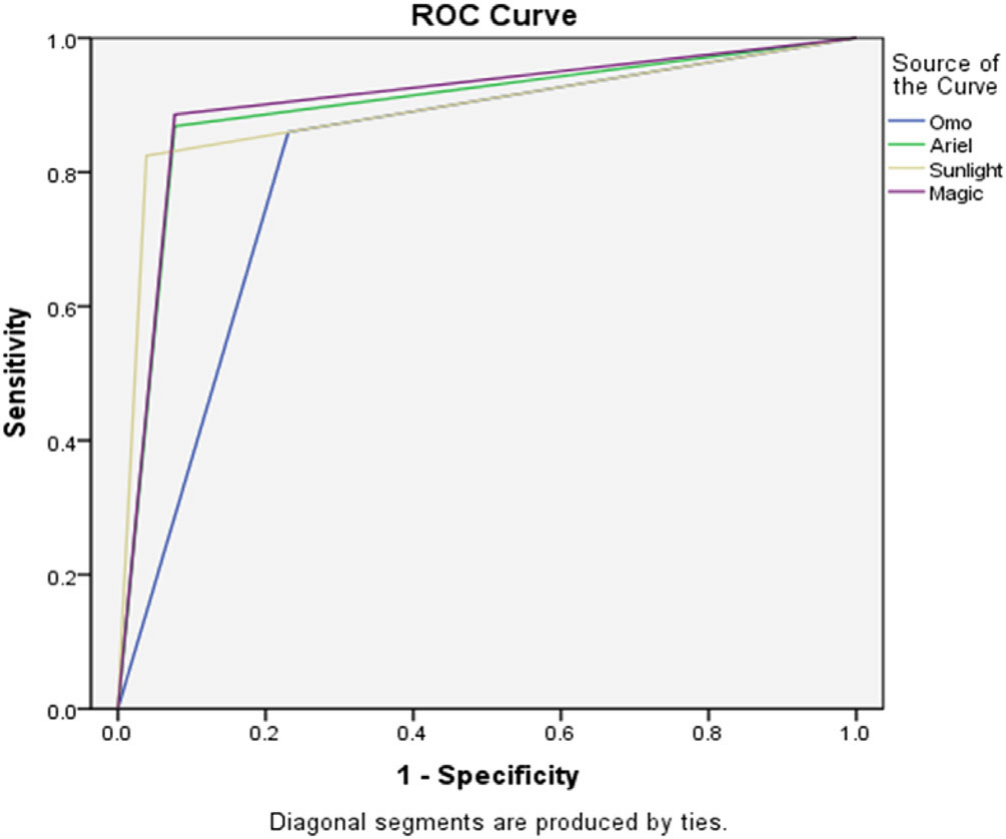
ROC analysis of the locally available washing detergents.

The data was captured using Microsoft Excel and transferred to SPSS software version 25 for analysis. Initially, univariate analyses were performed on all herd and animal level variables. Variables with a *p* value of < 0.25 on likelihood ratio Chi‐square test were included in multivariable models. A binary logistic regression model was designed to investigate individual cow level risk factors associated with subclinical mastitis. Fleiss kappa coefficient was used to determine the inter‐later reliability between the detergents and the commercial CMT reagent. Receiver–operator curve analysis was then done to determine the performance of each washing detergent as compared to the commercial CMT reagent (Figure 1). The area under the curve (AUC) for each of the washing detergents was determined (Akobeng 2007).

## 3 Results

### 3.1 Descriptive Statistics

Out of the 140 cows sampled, 43.6% were classified as young (3–5 years), 37.1% were classified as mature (6–9 years) and 19.3% classified as old (above 9 years). A total of 70.7% of the sampled cows were Friesians, 16.4% were Friesian‐Ankole crosses, 7.9% other exotic breeds while 5% were local zebu cows. Primiparous accounted for 21.4% and multiparous cows were 78.6% of the lactating cattle sampled.

Cows in early lactation were 29.3%, 32.1% in mid‐lactation while 38.6% were in late lactation. Of the cows sampled, 25.7% had a daily milk yield of up to 7 L, 49.3% had a daily milk yield of > 7–14 L while 25% had above 14 L. Drying off was done in 1 month to calving in 19.3% of the cows 70% were dried off 2 months pre‐calving whereas 10.7% were dried off 3 months pre‐calving.

At the time of milking, 36.4% of the cows had clean udders, 42.1% had slightly dirty udders and 16.4% had moderately dirty udders while 5% of the cows had very dirty udders.

There was a significant variation in the breed of the sampled cows (*p* < 0.001) with more Friesian cows sampled. There was a significant (*p* < 0.001) difference in the parity of animals sampled with more cows being multiparous as compared to primiparous. There was a significant (*p =* 0.001) difference in milk yield, with the majority of the cows producing > 7–14 L per day.

There was a significant (*p* = 0.001) difference in the duration of the dry off period with most cows being dried off 2 months before their expected calving date. The variation in the Udder Cleanliness score was also significant as there were more cows with slightly dirty and clean udders as compared to those which were very dirty (*p* = 0.001). Furthermore, there was a significant variation in the ages of the sampled cows with more younger and mature cows sampled than older ones (*p* = 0.001). Table 1 shows the detailed characteristics of the sampled cows.

**TABLE 1:** Characteristics of the sampled lactating cows.

Variable	Category	Frequency	Percentage (%)	Chi‐square	*p* value
Age	Young (3–5 years)	62	44.3	13.7	0.001*
	Mature (> 5–9 years)	51	36.4		
	Old (> 9 years)	27	19.3		
Breed	Friesian‐Ankole cross	27	19.3	148.6	< 0.001*
	Friesian	96	68.6		
	Local Zebu	6	4.3		
	Other exotic breeds	11	7.9		
Parity	Primiparous	31	22.1	43.5	< 0.001*
	Multiparous	109	77.1		
Lactation stage	Early lactation	39	27.9	3.7	0.157
	Mid lactation	57	40.7		
	Late lactation	44	31.4		
Milk yield	≤ 7 litres	43	30.7	16.7	< 0.001*
	>7–14 litres	68	48.6		
	Above 14 litres	29	20.7		
Dry off	1 month pre‐calving	27	19.7	86.2	< 0.001*
	2 months pre‐calving	98	70		
	3 months pre‐calving	15	10.7		
Udder Cleanliness score	Clean	51	36.4	50.3	< 0.001*
	Slightly dirty	59	42.1		
	Dirty	23	16.4		
	Very dirty	7	5		

### 3.2 Prevalence and Cow Level Risk Factors of Sub Clinical Mastitis in Urban and Peri‐Urban Cattle in Kampala

Of the 140 cows sampled, 114 were positive for subclinical mastitis giving a prevalence of 81.4%. There was a significant (*p* = 0.006) difference in mastitis prevalence across breeds, being higher in Friesians (Odds ratio = 8.047) followed by other exotic breeds (Odds ratio = 4.995) and Friesian‐Ankole crosses (Odds ratio = 1.395) as compared to the local zebu cows (Odds ratio = 1). Primiparous cows were also found to be less likely (Odds ratio = 0.189) to have subclinical mastitis as compared to multiparous cows (Odds ratio = 1) as shown in Table 2.

**TABLE 2:** Multivariate analysis of individual cow level risk factors for subclinical mastitis.

Variable	Category	S.E	*p* value	OR	95% CI	
					Lower	Upper
**Breed**			0.006*			
	Friesian	0.943	0.027	8.047	1.3	51
	Other exotic breeds	1.209	0.183	4.995	0.5	53.4
	Friesian‐Ankole	0.973	0.733	1.395	0.2	9.4
	Local zebu (Ref)			1		
**Parity**	Primiparous	0.517	0.001	0.189	0.07	0.5
	Multiparous (Ref)			1		

### 3.3 Performance Testing of Different Domestic Detergents on the Detection of Subclinical Mastitis

The inter‐later reliability analysis of the performance of different domestic reagents was carried out and the Fleiss kappa coefficient was determined and found to be *r* = 0.556 (95% confidence interval: 0.504–0.608) implying a moderate to good reliability of the domestic reagents used in Uganda when compared with the CMT reagent as shown in Table 3, 4.

**TABLE 3:** Fleiss kappa inter‐later reliability analysis of the detergents with CMT.

	Kappa	Asymptotic standard error	*Z*	*p* value	Lower 95% asymptotic CI bound	Upper 95% asymptotic CI bound
Overall	0.556	0.027	20.806	0.000*	0.5	0.6

*Note*: Areas under the receiver operating characteristics curve were recorded 0.814, 0.896, 0.893 and 0.905 for 6% Omo, 5% Ariel, 5% Sunlight and 6% Magic respectively as shown in Table 4.

**TABLE 4:** Area under the curve from the ROC analysis.

				95% Confidence interval	
	AUC	Standard error	Sig.	Lower bound	Upper bound
6% Omo	0.814	0.052	0.000	0.7	0.9
5% Ariel	0.896	0.036	0.000	0.8	1
5% Sunlight	0.893	0.031	0.000	0.8	1
6% Magic	0.905	0.035	0.000	0.8	1
CMT (Reference test)	1.000				

## 4 Discussion

From this study, the prevalence of subclinical mastitis in urban and peri‐urban Kampala was found to be at 81.4%. This prevalence was comparable to the prevalence of 86.2% and 90% that was found in the same study area (Kampala) in 2011 and 2013 by other researchers (Abrahmsén et al. 2014; Björk 2013). This was however higher than the national average of 75.71% from a systematic review in 2025 (Muhwezi et al. 2025) and higher than the prevalence found in other parts in Uganda like Wakiso district with 76% in 2023 (Kakooza et al. 2023), Kiboga district 87.9% (Kasozi et al. 2014) and Kiruhura district 76.1% (Ssajjakambwe et al. 2017). This high prevalence is because most of the sampled animals were kept under the zerograzing system where transmission of mastitis from the dirty environment is common. The high prevalence could also be because the study was carried out just at the end of the rainy season in Uganda (May to June) which is normally associated with high rate of udder infections.

The high prevalence was probably because there were significant variations in the characteristics of the cows sampled for the study. There were more Friesians than other breeds used in the study, more multiparous cows were also selected as compared to primiparous cows. There was also a significant variation in the ages of the cows with a combined more mature and older cows selected as compared to younger ones. There was also a significant variation in the daily milk yield with more high milk producing selected for the study and fewer poor milk producers. This would probably have had a combined effect of the high prevalence of subclinical mastitis in urban and peri‐urban Kampala (Abrahmsén et al. 2014; Biffa et al. 2005; Björk 2013; Tolosa et al. 2013; Mekonnen et al. 2017). The high prevalence could also be attributed to the number of cows in late lactation which could increase the number of false positives in the study.

At individual cow level, the risk for subclinical mastitis increased with parity (*p* < 0.001) and breed (*p* = 0.006) of cattle. Higher prevalence was found in the Friesian (Odds ratio = 8.047; 95% confidence interval: 1–51), other exotic breeds (Odds ratio = 4.995; 95% confidence interval: 0.5–53) than the Friesian‐Ankole crosses (Odds ratio = 1.395; 95% confidence interval: 0.2–9.4) as compared to the local zebu cows (Odds ratio = 1). Local zebu breeds have been found to be more resistant to mastitis than the exotic breeds. This is probably due to the fact that they are very poor milk producers hence their low susceptibility to subclinical mastitis. Previous studies confirmed that the Holstein Friesian breed is more susceptible to udder infection; particularly in areas where hygienic conditions are poor and treatment of mastitis cases is not well managed (Moges et al. 2012). The occurrence of mastitis may be influenced by some heritable characteristics such as milk production capacity, teat structure and udder conformation (Schutz 1994).

Primiparous cows were also found to be less likely to have subclinical mastitis (Odds ratio = 0.189; 95% confidence interval: 0.07–0.5) as compared to multiparous cows (Odds ratio = 1). This finding agreed with other studies that found an increasing prevalence with parity (Abrahmsén et al. 2014; Rahman et al. 2009). This was due to the fact that multiparous cows are exposed to infection for longer periods with subsequent lactations. The efficacy of the defence mechanism of the mammary gland also decreases progressively with the increase in the number of lactations. This is probably because the teat canal in older animals is more dilated and or it remains partially open permanently due to years of repeated milking (Madut et al. 2009).

The Fleiss kappa coefficient, *r* = 0.556 (95% confidence interval: 0.5–0.60) implying a moderate to good reliability of the domestic reagents along with the CMT reagent. This showed that all the domestic washing detergents used in the study can be used to detect for subclinical mastitis in cattle. This could be due to the shared detergency properties of both the CMT reagent and the domestic reagent. This shows that the domestic reagents used in the study have enough anionic surfactants to combine with milk such that they can dissolve the walls of the somatic cells in the milk causing them to lyse (Leach et al. 2008). The lysis of somatic cells by these anionic surfactants leads to precipitation of the DNA and proteins contained in the cells forming the gel formation which is used to score for subclinical mastitis (Blum et al. 2000).

The accuracy of the detergents determined by summarizing the sensitivity and specificity using the AUC after carrying out receiver operating characteristics (ROC) analysis were recorded 0.814, 0.896, 0.893 and 0.905. An AUC of 0.8–0.9 represent a good measure while an AUC of 0.9–1 represent an excellent/high accurate test (Akobeng 2007). Basing on this measure, 6% Omo, 5% Ariel and 5% Sunlight had a good measure of accuracy while 6% Magic had an excellent measure of accuracy. The failure of these detergents to attain a an ACU of 1 which represents excellent accuracy means that, they can have false positives especially in animals that are pregnant and dried off or those that have recently given birth thus farmers should double check cattle that form weak coagulation (false positive animals) in such situations.

The ability of the domestic washing detergents to detect mastitis is due to the presence of anionic surfactants in their composition which is the main ingredient in the CMT reagent. The largest composition of laundry detergents is surfactants of which the anionic surfactants are of the highest proportion due to their ability to remove dirt. The CMT reagent and the domestic detergents therefore had a good to excellent accuracy due to the shared detergence properties of the reagents (Muhammad et al. 2010).

The difference in the area under the receiver operator curve of the different domestic reagents is probably due to the differences in the concentration the anionic surfactants in the individual detergents. Although the specific concentration of the surfactants in the locally available domestic detergents used in the study were not readily available to the public, it was shown in a different study that the sensitivity and specificity of the detergents in detecting subclinical mastitis in cattle was higher in detergents which had a high concentration of anionic surfactants. It was further shown that the detergents which had less anionic surfactant had lower sensitivity and specificity to screen for subclinical mastitis (Leach et al. 2008).

Although locally available detergents showed a comparable diagnostic accuracy to CMT, further studies are needed on their cost per test, ease of preparation, environmental safety and the training requirements before they can be rolled out for use.

## Author Contributions

All authors contributed substantially to this research in different sections from conceptualization, funding, methodology, data collection, data curation, data analysis, drafting the manuscript, reviewing the manuscript

## Ethics Statement

This research was approved by the school of veterinary medicine and animal resources (SVAR), Makerere University review committee for undergraduate students’ research projects and was allowed to be carried out since it posed minimal risk to animals and did not involve humans and thus did not require additional clearance from national animal and human user committees.

## Conflicts of Interest

The authors declare no conflicts of interest.

## Peer Review

The peer review history for this article is available at https://www.webofscience.com/api/gateway/wos/peer‐review/10.1002/vms3.70635.

## Data Availability

Data on this research can be got from the corresponding author on request free of charge and can be published on public data bases.
